# Metabolomic analysis of *Drosophila melanogaster* larvae lacking pyruvate kinase

**DOI:** 10.1093/g3journal/jkad228

**Published:** 2023-10-04

**Authors:** Yasaman Heidarian, Jason P Tourigny, Tess D Fasteen, Nader H Mahmoudzadeh, Alexander J Hurlburt, Travis Nemkov, Julie A Reisz, Angelo D’Alessandro, Jason M Tennessen

**Affiliations:** Department of Biology, Indiana University, Bloomington, IN 47405, USA; Department of Biology, Indiana University, Bloomington, IN 47405, USA; Department of Biology, Indiana University, Bloomington, IN 47405, USA; Department of Biology, Indiana University, Bloomington, IN 47405, USA; Department of Biology, Indiana University, Bloomington, IN 47405, USA; Department of Biochemistry and Molecular Genetics, Anschutz Medical Campus, University of Colorado School of Medicine, Aurora, CO 80045, USA; Department of Biochemistry and Molecular Genetics, Anschutz Medical Campus, University of Colorado School of Medicine, Aurora, CO 80045, USA; Department of Biochemistry and Molecular Genetics, Anschutz Medical Campus, University of Colorado School of Medicine, Aurora, CO 80045, USA; Department of Biology, Indiana University, Bloomington, IN 47405, USA

**Keywords:** *Drosophila melanogaster*, metabolism, glycolysis, pyruvate kinase, metabolomics

## Abstract

Pyruvate kinase (Pyk) is a rate-limiting enzyme that catalyzes the final metabolic reaction in glycolysis. The importance of this enzyme, however, extends far beyond ATP production, as Pyk is also known to regulate tissue growth, cell proliferation, and development. Studies of this enzyme in *Drosophila melanogaster* are complicated by the fact that the fly genome encodes 6 Pyk paralogs whose functions remain poorly defined. To address this issue, we used sequence distance and phylogenetic approaches to demonstrate that the gene *Pyk* encodes the enzyme most similar to the mammalian Pyk orthologs, while the other 5 *Drosophila* Pyk paralogs have significantly diverged from the canonical enzyme. Consistent with this observation, metabolomic studies of 2 different *Pyk* mutant strains revealed that larvae lacking Pyk exhibit a severe block in glycolysis, with a buildup of glycolytic intermediates upstream of pyruvate. However, our analysis also unexpectedly reveals that pyruvate levels are unchanged in *Pyk* mutants, indicating that larval metabolism maintains pyruvate pool size despite severe metabolic limitations. Consistent with our metabolomic findings, a complementary RNA-seq analysis revealed that genes involved in lipid metabolism and protease activity are elevated in *Pyk* mutants, again indicating that loss of this glycolytic enzyme induces compensatory changes in other aspects of metabolism. Overall, our study provides both insight into how Drosophila larval metabolism adapts to disruption of glycolytic metabolism as well as immediate clinical relevance, considering that Pyk deficiency is the most common congenital enzymatic defect in humans.

## Introduction

Pyruvate kinase (Pyk; E.C. 2.7.1.40) is an ancient enzyme that catalyzes the final reaction of glycolysis (phosphoenolpyruvate + ADP + Pi to pyruvate + ATP) in organisms that span all 3 domains of life (Hattori et al. 1995; Muñoz and Ponce 2003; Falb et al. 2008; Johnsen et al. 2019; Schormann et al. 2019). Since the Pyk-catalyzed reaction is essentially irreversible under normal cellular conditions (Schormann et al. 2019), this enzyme controls a key rate-limiting, “pay-off” glycolytic step that controls ATP production, and dictates the rate at which glycolytic intermediates enter the metabolic pathways that branch off of glycolysis. One consequence of this regulation is that Pyk controls the size of the 3-phosphoglycerate pool and the flux of this metabolite into serine synthesis, thus indirectly influencing redox balance and nucleoside anabolism via production of the amino acids cysteine (de novo glutathione synthesis) and glycine (one-carbon metabolism for de novo purine synthesis) (for examples, see Maddocks et al. 2013 and Zhang, Lai et al. 2017). This central role for Pyk in coordinating cellular metabolism has become the topic of great interest in models of human disease, where changes in Pyk activity can influence cell fate decisions by balancing the flux of glycolytic metabolites between energy production and the synthesis of metabolites involved in chromatin remodeling, one-carbon metabolism, and biomass production (Israelsen and Vander Heiden 2015; Hsu and Hung 2018).

The importance of Pyk in human health is clear from the myriad of diseases that are caused by altered activity of the 2 human paralogs, Pyruvate Kinase, Liver and Red Blood Cell (PKLR) and Pyruvate Kinase, Muscle (PKM). For example, mutations in the *PKLR* gene result in a common congenital disorder known as Pyk deficiency (PKD) (Fattizzo et al. 2022; Luke et al. 2023). Individuals with PKD experience a wide spectrum of clinical and biochemical symptoms and have a highly variable prognoses that depend on environmental factors, early interventions, and the severity of the causal mutations (Dolan et al. 2002; Grace et al. 2015). Similarly, changes in splicing of PKM transcripts are well-documented in a number of disease states, with a switch from the PKM1 isoform towards the PKM2 isoform associated with pulmonary hypertension (Caruso et al. 2017; Zhang, Wang et al. 2017), sporadic Alzheimer's disease (Traxler et al. 2022), and enhancement of the Warburg effect in cancer cells (Israelsen and Vander Heiden 2015). Finally, population genetic studies support a model in which *PKLR* mutations are protective against malaria (Min-Oo et al. 2007; Ayi et al. 2008; Machado et al. 2012; van Bruggen et al. 2015), suggesting that partial inhibition of the enzyme could be exploited towards antimalarial efforts. Basic studies of PYK function in the context of endogenous metabolic networks thus hold the potential to inform better interventions for a wide range of human diseases.

The fruit fly *Drosophila melanogaster* is a powerful model for studying the function of metabolic enzymes within the context of both normal physiology and human disease models (Drummond-Barbosa and Tennessen 2020; Kim et al. 2021). In this regard, previous studies of *Drosophila* Pyk (FBgn0267385) have demonstrated roles for this enzyme in fly development and behavior, with reduced *Pyk* expression being associated with defects in muscle and wing development, neuron and glial function, and olfactory memory (Tixier et al. 2013; Volkenhoff et al. 2015; Wu et al. 2018; Spannl et al. 2020). However, the effects of decreased Pyk activity in the fly have not been examined at a metabolic level. Moreover, the fly genome encodes 6 predicted Pyk paralogs (Pyk, CG7069, CG2964, CG7362, CG11249, and CG12229, Garapati et al. 2019; Gramates et al. 2022), thus highlighting a need to characterize the in vivo functional potential of individual enzymes within this gene class.

Here, we use sequence identities and phylogenetics in combination with metabolomics and transcriptomics to analyze *Pyk* (FBgn0267385), which encodes the most abundantly and widely expressed member of the *D. melanogaster* Pyk family (Graveley et al. 2011). Our studies indicate that the gene *Pyk* encodes the *Drosophila* enzyme most similar to human PKM and PKLR. Subsequent metabolomic analysis of 2 *Pyk* mutant strains reveals a severe block in glycolysis, with a buildup of intermediates immediately upstream of Pyk and a depletion of lactate, 2-hydroxyglutarate (2HG), and the amino acid proline. Moreover, loss of Pyk activity also induces a significant upregulation of genes involved in lipid metabolism and protease activity. Overall, our study validates the presumed function of Pyk within *Drosophila* metabolism and identifies the transcriptional and metabolic networks that respond to loss of Pyk activity during larval development.

## Methods

### *Drosophila* husbandry and larval collection

Fly stocks were maintained on Bloomington *Drosophila* Stock Center media at 25°C. Larvae were collected on molasses agar plates as previously described (Li and Tennessen 2017, 2018). Briefly, 50 virgin females and 25 males of the appropriate genotypes were placed in 6-ounce plastic bottles (Genesee Scientific; Cat #: 32-130) with holes punched in the side using a 20-gauge needle. A 35-mm molasses agar plate (Corning 353001) with a smear of yeast paste on the surface was taped in place in the mouth of the bottle and the bottle inverted and placed in a 25°C incubator. The molasses agar/yeast plate was replaced at least once per day. For sample collection, eggs were collected on the molasses agar/yeast plate for 6 hours, individual plates were placed inside a 60-mm cell culture dish, and the collected embryos were placed in a 25°C incubator and allowed to develop for 48 hours. Larvae were collected at the early L2 stage for subsequent metabolomic analysis. Larval age was verified based on the trachea structure and mouthhook morphology. Note that male and female larvae are exceedingly difficult to distinguish at this stage of development, thus we used a mix of both sexes in our analysis.

### Generation of *Pyk* alleles using CRISPR/Cas9

The *Pyk* deletions *Pyk^23^* and *Pyk^31^* were generated using a previously described approach for CRISPR/Cas9 mutagenesis (Gratz et al. 2013; Sebo et al. 2014). Briefly, oligos containing gRNA sequences #1 (5′-GTGCCCCATGTGCGTCTGTC-3′) and #2 (5′-GCTGCTGGAGGCAGGTCCGA-3′) were inserted into pU6-BbsI-chiRNA (DGRC Stock #1362), and the resulting plasmids were independently injected into BDSC Stock #52669 (*y^1^ M{RFP[3xP3.PB] GFP[E.3xP3]=vas-Cas9.S}ZH-2A w^1118^*) by Rainbow Transgenics (Camarillo, CA). Injected females and F1 progeny were crossed to w*; ry^506^*Dr*^1^*/TM3, P{Dfd-GMR-nvYFP}3, Sb^1^*. Putative F2 *Pyk^Δ^/TM3, P{Dfd-GMR-nvYFP}3, Sb^1^* siblings were mated and the F3 generation screened for animals lacking the *TM3* balancer, indicating the presence of a lethal mutation. Any strain that failed to generate homozygous mutant adults was crossed with *Pyk^61^* mutants and the F1 progeny analyzed for failure to complement. Mutant strains that failed to complement *Pyk^61^* were further analyzed for the presence of *Pyk* mutations. *Pyk^23^* was isolated from injections using gRNA#1, and *Pyk^31^* was isolated from injections using gRNA#2. The deletions were identified by amplifying and sequencing a region of the *Pyk* gene using oligos 5′-CACGCACTTTGTTTACATCAGC-3′ and 5′-GCACCAGTCCACGGTAGAGA-3′.

### Generation of *Pyk* alleles using p-element excision

Both the *Pyk^60^* and *Pyk^61^* alleles were generated for this study using standard techniques for imprecise p-element excision (Robertson et al. 1988; Bellen et al. 2004). Briefly, virgin females containing the transposon insertions in the strains *Pyk^DG05605^/TM3, Sb*^1^*, Ser^1^* and *Pyk^EY10213^/TM3, Sb*^1^*, Ser^1^* (BDSC stock 20088) were independently crossed with *ry506 p{Δ2-3}99B* males. F1 virgin females lacking the TM3 balancer were crossed with w*; ry^506^*Dr*^1^*/TM3, P{Dfd-GMR-nvYFP}3, Sb^1^* males (BDSC stock 23231). F2 males with white eyes were then crossed to w*; ry^506^*Dr*^1^*/TM3, P{Dfd-GMR-nvYFP}3, Sb^1^* females, and F3 *Pyk^Δ^/TM3, P{Dfd-GMR-nvYFP}3, Sb^1^* siblings were used to establish mutant lines. The *Pyk^60^* allele was generated by the imprecise excision of *Pyk^EY21058^*, and the *Pyk^61^* deletion was generated by the imprecise excision of *Pyk^DG05605^*. Endpoints of the deletions were mapped using a PCR-based approach.

The *Pyk^61^* deletion was sequenced by isolating homozygous mutant larvae, amplifying across the deletion using the oligos 5′-GATTTCCTTCAGAGCATTTGCGTC-3′ and 5′-TTCACCGTGCAGCAAGACATC-3′ and sequencing the resulting PCR product. The *Pyk^61^* deletion is 6400 bp long starting with the sequence 5′-GATGCCTTTGTTGCCGCCCT-3′ and ending with the sequence 5′-GTATGAACTTCTCTCACGGC-3′ (3R:22373250…22368775; based on the *D. melanogaster* reference genome release 6.53), and includes an 18-bp insertion of the sequence 5′-AAGTTCAAGTTCTGGATT-3′ (see File S1). As illustrated below, *Pyk^61^* deletes the entire *Pyk*-coding region as well as the first-coding exons of both the upstream and downstream neighboring genes (*Polr3f* and *CG7069*).

The *Pyk^60^* allele represents a large deletion or rearrangement between the sequences 5′-CTTCTGTTCTATCCGATTGCCGG-3′ and 5′-TTGACGCGCTCATTGGGTTTC-3′ (these sequences represent the oligos closest to the insertion site that can be used to successfully generate PCR products that flank the lesion). However, despite many attempts, we were unable to produce a PCR product spanning the lesion. The *Pyk^prec^* allele is a precise excision of *Pyk^DG05605^*. For all analyses, the *Pyk^60^/TM3, P{Dfd-GMR-nvYFP}3, Sb^1^* and *Pyk^61^/TM3, P{Dfd-GMR-nvYFP}3, Sb^1^* strains were crossed and trans-heterozygous *Pyk^60/61^* mutant larvae were selected for based on the absence of YFP.

Since the transposon excision events that produced the *Pyk^60^* and *Pyk^61^* deletion alleles also disrupted neighboring genes, we further analyzed the effects of these deletions on expression of neighboring genes. When these deletions are placed in *trans*, the resulting heterozygote lacks sequence corresponding to nearly the entire *Pyk*-coding region and the 5′ exons of the neighboring *Pyk* homolog *CG7069*. Northern blot analysis of trans-heterozygous L2 larvae revealed that *Pyk^60/61^* larvae fail to accumulate detectable *Pyk* mRNA transcripts (Supplementary Fig. 1). The mRNA of neighboring genes *Polr3F* and *CG18596*, however, was present at similar levels in both *Pyk^Prec^* and *Pyk^60/61^*, indicating that the phenotypes arising from the *Pyk* mutant background are specific to loss of *Pyk* expression. Of note, the second pyruvate kinase homolog disrupted by these deletions, *CG7069*, is primarily expressed in adult testis, and transcripts are present at very low or undetectable levels during the embryonic and larval stages (see the RNA-seq study described below and FlyBase, Chintapalli et al. 2007; Robinson et al. 2013; Brown et al. 2014; Gramates et al. 2022). Since *Pyk^60/61^* larvae die prior to the L3 stage, the effects of these deletions on *CG7069* expression could not be examined.

### Northern blot analysis

RNA was extracted from L2 larvae with TriPure isolation reagent, and northern blot analysis was conducted as previously described (Karim and Thummel 1991; Sullivan and Thummel 2003). The following PCR oligos were used to generate northern blot probes: *Pyk*: 5′-GCTGACCACCAACAAGGAAT-3′ and 5′-GCACCAGTCCACGGTAGAGA-3′; *Polr3F*: 5′-ACCAACGATGACCTGACCAAG-3′ and 5′-ATTGTTTCCAGATCGGCCTCC-3′; and *rp49*: 5′-ACAAATGTGTATTCCGACCACG-3′ and 5′-TCAAGATGACCATCCGCCCAG-3′.

### Phylogenetic analysis of Pyk orthologs and paralogs

Protein sequences for each of the Pyk homologs were retrieved from FlyBase (Gramates et al. 2022), ZFIN (Bradford et al. 2022), WormBase (Davis et al. 2022), NCBI RefSeq (O’Leary et al. 2016), dictyBase (Fey et al. 2013), and UniProt (Bateman et al. 2023). The longest verified isoform of each was selected for multiple sequence alignment (MSA; see Supplementary Table 1 for protein sequence information), except for mosquito which was a predicted entry (Petchampai et al. 2019). The MSA was generated via MAFFT v7.520 (Katoh and Standley 2013) using the E-INS-I algorithm (command: “mafft –maxiterate 1000 –thread 4 –genafpair [FASTA_in] > [MSA_out]”). For the sequence identity and similarity heatmaps, the resulting MSA was analyzed using the “seqidentity()” function of the Bio3D R package (Grant et al. 2006) to extract pairwise matrices, which were visualized in R with corrplot (Wei and Simko 2021).

For the phylogenetic analysis, we used the same MSA to reconstruct the Pyk evolutionary tree with IQ-TREE2, applying the WAG model of protein substitution (Whelan and Goldman 2001; Nguyen et al. 2015; Hoang et al. 2018; Minh et al. 2020), run with the command as follows: “iqtree2 -s [MSA_in] –redo -B 1000 -T 4 -m WAG –prefix [TREE_out]”. The resulting phylogeny was rooted to *Dictyostelium discoideum*, formatted, and visualized using FigTree v1.4.4 (Rambaut 2018).

### Triglyceride assays and Nile red staining

Triglyceride levels in *Pyk* control and mutant L2 larvae were measured as previously described (Tennessen et al. 2014). Briefly, synchronized populations of embryos were collected for 4 hours on a molasses agar plate with yeast paste smeared on the surface, as described (Li and Tennessen 2017). Embryos were allowed to hatch on the surface of the collection plate, and larvae were reared on the same plate in a 25°C incubator. Sixty hours after egg-laying, heterozygous controls and homozygous mutant larvae were identified based on lack of GFP expression from the *TM3, P{Dfd-GMR-nvYFP}3, Sb^1^* balancer chromosome. Twenty-five larvae of the appropriate genotypes were then collected in 1.5-mL microfuge tubes, washed 3 times with phosphate-buffered saline pH 7.0 (PBS), homogenized in 100 µL of cold PBS + 0.05% Tween 20 (PBST), and heat-treated for 10 minutes at 70°C. The resulting homogenate was assayed for triglyceride and soluble protein levels as previously described (Tennessen et al. 2014).

Nile red staining was conducted on L2 fat bodies as previously described (Grönke et al. 2005). Briefly, dissected tissues were fixed with 4% paraformaldehyde in PBS for 30 minutes and washed with PBST 3 times for 5 minutes each, and then incubated for 1 hour at room temperature in a 1:1,000 dilution of Nile red stock (10 mg/mL in acetone) in PBS. Tissues were washed 3 times for 5 minutes each in PBST, mounted in Vectashield (Vector Laboratories), and imaged on a Leica SP8 Confocal at 568 nm.

Measurements for total area of lipid droplets per total area of tissue section and individual lipid droplet area were conducted using ImageJ as previously described (Musselman and Kühnlein 2018). A threshold was manually set for the selected image that maximized droplet visibility and minimized background. For total area of lipid droplets, boundaries were drawn around the border of the fat body tissue shown in the image, and the “measure” function was used to produce measurements for “%area” for each of 3 representative images for both *Pyk^23/31^* and *Pyk^23/+^*. For measurements of individual lipid droplet area, the “analyze particles” function was used to produce area measurements for lipid droplets from 3 representative images.

### Gas chromatography mass spectrometry (GC-MS)-based metabolomics

*Pyk* mutant and control larvae were collected at the early L2 stage (∼48 hours after egg-laying at 25°C) as previously described (Li and Tennessen 2018). All samples collected for analysis represent biological replicates obtained from independent mating bottles and each sample contained 25 larvae. Analysis of the *Pyk^60^* and *Pyk^61^* alleles was conducted by the University of Utah metabolomics core facility as previously described (Cox et al. 2017). *Pyk^23^*^/31^ mutants and *Pyk^23/+^* control samples were analyzed by the University of Colorado Metabolomics core facility. Data were normalized to sample mass and internal standards.

### Ultra high-pressure liquid chromatography–mass spectrometry (UHPLC-MS)-based metabolomics

Ultra high-pressure liquid chromatography–mass spectrometry (UHPLC-MS) metabolomic analyses were performed at the University of Colorado Anschutz Medical Campus, as previously described (Nemkov et al. 2019). Briefly, the analytical platform employs a Vanquish UHPLC system (Thermo Fisher Scientific, San Jose, CA, USA) coupled online to a Q Exactive mass spectrometer (Thermo Fisher Scientific, San Jose, CA, USA). The (semi)polar extracts were resolved over a Kinetex C18 column, 2.1 × 150 mm, 1.7-µm particle size (Phenomenex, Torrance, CA, USA) equipped with a guard column (SecurityGuard Ultracartridge—UHPLC C18 for 2.1-mm ID Columns—AJO-8782—Phenomenex, Torrance, CA, USA) using an aqueous phase (A) of water and 0.1% formic acid and a mobile phase (B) of acetonitrile and 0.1% formic acid for positive ion polarity mode, and an aqueous phase (A) of water:acetonitrile (95:5) with 1-mM ammonium acetate and a mobile phase (B) of acetonitrile:water (95:5) with 1-mM ammonium acetate for negative ion polarity mode. The Q Exactive mass spectrometer (Thermo Fisher Scientific, San Jose, CA, USA) was operated independently in positive or negative ion mode, scanning in Full MS mode (2 μscans) from 60 to 900 m/z at 70,000 resolution, with 4-kV spray voltage, 45 sheath gas, and 15 auxiliary gas. Calibration was performed prior to analysis using the Pierce Positive and Negative Ion Calibration Solutions (Thermo Fisher Scientific).

### Statistical analysis of metabolomics data

Both metabolomics datasets were analyzed using Metaboanalyst 5.0 (Pang et al. 2021), with data first preprocessed using log normalization and Pareto scaling.

### RNA-seq analysis

RNA from *Pyk^23/+^* heterozygous controls and *Pyk^23/31^* mutants was extracted from L2 larvae with TriPure isolation reagent and further purified using a Qiagen RNeasy kit (Catalog #74004). RNA-seq was performed on 3 whole-fly biological replicates for *Pyk^23/+^* heterozygous controls and *Pyk^23/31^* mutants. Samples were paired-end sequenced on the NextSeq 550 platform 75 cycles to a depth of 15–20 million reads each, using standard Illumina TruSeq Stranded mRNA libraries, at the IU Center for Genomics and Bioinformatics.

RNA-seq read quality was assessed with FastQC (Andrews 2010) and MultiQC (Ewels et al. 2016); raw reads were not trimmed or filtered. Reads were pseudo-aligned and quantified using Kallisto v0.46.0 (Bray et al. 2016), the *D. melanogaster* BDGP6.32 reference assembly and annotation retrieved through Ensembl (Cunningham et al. 2022), and the gffread utility from the Cufflinks suite to generate the transcriptome from the assembly and annotation (Trapnell et al. 2010).

Differential expression analysis was performed with tximport (https://doi.org/10.12688/f1000research.7563.1) and DESeq2 v1.30.1 (Love et al. 2014) running in RStudio v1.4.1717 on R v4.0.4. Genes with average expression below 2 counts per sample (i.e. 12 counts per row) were filtered out before determining genes with significant expression difference based on a Wald test adjusted *P*-value ≤ 0.05 and an absolute log fold change ≥ 1. Subsequent analysis and visualization were performed in R using a variety of tools and packages. Correlation plots were generated using base R and the corrplot package v0.92 (Wei and Simko 2021). Principal components were analyzed and visualized with PCAtools v2.2.0 (Blighe and Lun 2022). Heatmaps, including clustering analysis performed by the hclust method (from base R {stats} package), were generated by pheatmap v1.0.12 (Kolde 2019).

Gene Ontology Analysis was carried out using the GOrilla tool for genes that were significantly (adjusted *P*-value < 0.05) upregulated or downregulated by more than 2-fold in *Pyk* mutants (Eden et al. 2009). Tissue enrichment analysis was conducted using default settings for the “Preferred tissue (modEncode RNA_seq)” tool in PANGEA version 1.1 beta (Hu et al. 2023).

### Pathway enrichment analysis

The list of genes and metabolites that were significantly altered in *Pyk^23/31^* mutants compared to *Pyk^23/+^* controls were analyzed using OmicsNet 2.0 (omicsnet.ca) (Zhou et al. 2022). Subnetworks were generated using the KEGG (Organism-specific) database followed by the “Minimum Network” option. Only those subnetworks containing both significantly altered genes and metabolites are described below.

## Results

### The *Drosophila* gene *Pyk* encodes the Pyk enzyme orthologous to human PKs

**Fig. 1.**
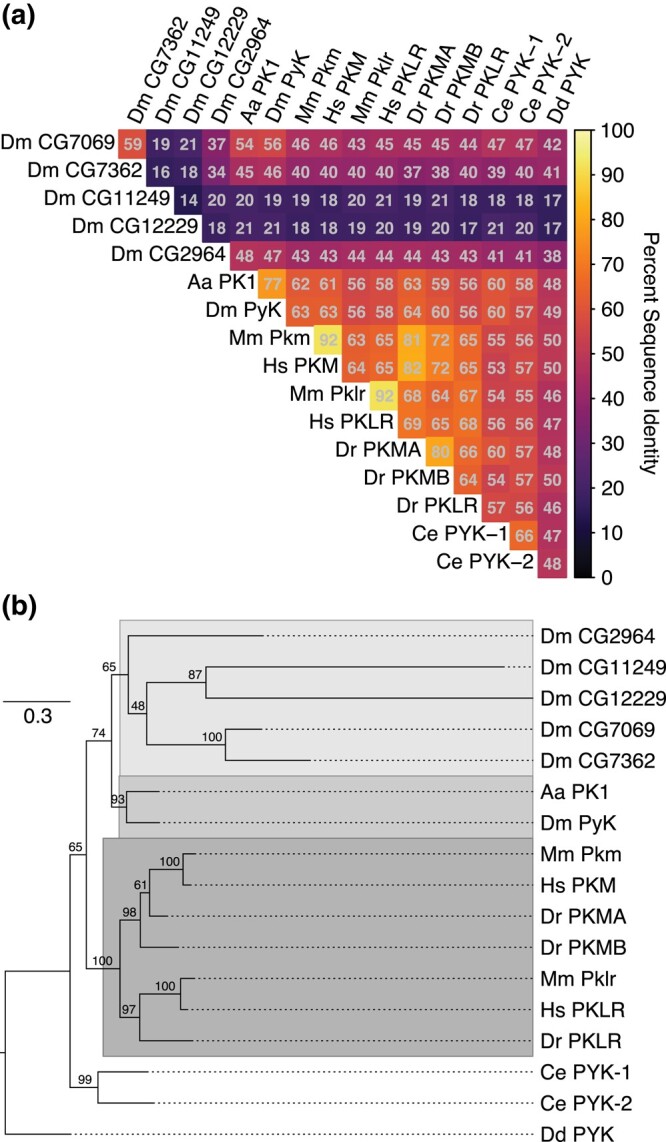
Sequence identity and phylogeny of Pyk homologs. a) Heatmap of pairwise sequence identities extracted from the multiple sequence alignment (MSA). b) Phylogenetic maximum likelihood Pyk gene tree. Ultrafast bootstrap support is shown on internal branches. The tree is rooted to *D*. *discoideum* homolog PYK as the outgroup. Species abbreviations: Aa, *A. aegypti*; Dm, *D. melanogaster*; Hs, *Homo sapiens*; Mm, *Mus musculus*; Ce, *C. elegans*; Dr, *D. rerio*; Dd, *D. discoideum*. See Supplementary Table 1 for the list of isoform-specific coding sequences (CDSs) used in the analysis.

The *Drosophila* genome harbors 6 genes predicted to encode pyruvate kinases (Garapati et al. 2019; Gramates et al. 2022). To better understand the relationships between the *Drosophila* Pyk paralogs and Pyk orthologs in other organisms, we compared their pairwise protein sequence identities and generated a phylogenetic gene tree to infer their evolutionary history (Fig. 1, Supplementary Table 1). Species examined included human and mouse, *Aedes aegypti*, *Caenorhabditis elegans*, *Danio rerio*, and *D. discoideum* as the outgroup for tree rooting (Supplementary Table 1). The pairwise sequence comparisons indicate that *Drosophila* Pyk shares strongest identity with that of the *A. aegypti* protein PK1 (XP_001649983.1), followed by those of its vertebrate orthologs, the worm homologs, and fly paralog CG7069 (Fig. 1a). The other putative paralogs show identity to a lesser extent, with 2 of the *Drosophila* proteins (CG11249 and CG12229) displaying 21% or less identity with all other analyzed Pyk orthologs (Fig. 1a). The sequence similarities, allowing for conservative residue substitutions, show a similar pattern (Supplementary Fig. 2). These results indicate that, at the sequence level, *Drosophila* Pyk is as, or more, similar to the *C. elegans* homologs than it is to 4 of the 5 other putative fly paralogs.

Sequence distances may not correspond to actual evolutionary or functional relationships, so we next sought to reconstruct the Pyk gene tree using a maximum likelihood model of protein evolution. The resulting phylogeny indicates that the *Drosophila* Pyk is sister to its *A. aegypti* ortholog (Fig. 1b), which compose a larger clade with the more distantly related *Drosophila* paralogs. This insect Pyk/PK1 clade is in turn sister to that of the vertebrate homologs, which share a common ancestor. We note generally strong ultrafast bootstrap support for the insect and vertebrate ortholog subclades, the worm paralogs, and 2 pairs of the fly paralogs, with weaker support for the fly paralog internal branches and the resolution between insects and vertebrates (Fig. 1b). While phylogenetically we cannot claim any further orthology between fly and vertebrate Pyks based on this tree, the divergence of the fly paralogs is readily apparent from their extended branch lengths.

*Aedes* PK1 was previously demonstrated to exhibit unique allosteric regulation when compared with mammalian enzymes (Petchampai et al. 2019). Given the high sequence similarity and close evolutionary relationship between these 2 dipteran proteins, our findings suggest that *Drosophila* Pyk likely functions more similarly to mosquito PK1 than to the mammalian enzymes. Thus, future studies of *Drosophila* Pyk should be hesitant to make direct comparisons between the fly enzyme and either of the mammalian enzymes, especially in the context of human disease models (see discussion below). Overall, our results support the hypothesis that the fly gene *Pyk* encodes the canonical glycolytic enzyme, which is consistent with previous studies demonstrating that *Pyk* is the most widely expressed member of the *D. melanogaster* Pyk gene class (Rust and Collier 1985; Chen et al. 2011; Graveley et al. 2011).

### *Pyk* mutants die during the second larval instar

**Fig. 2.**
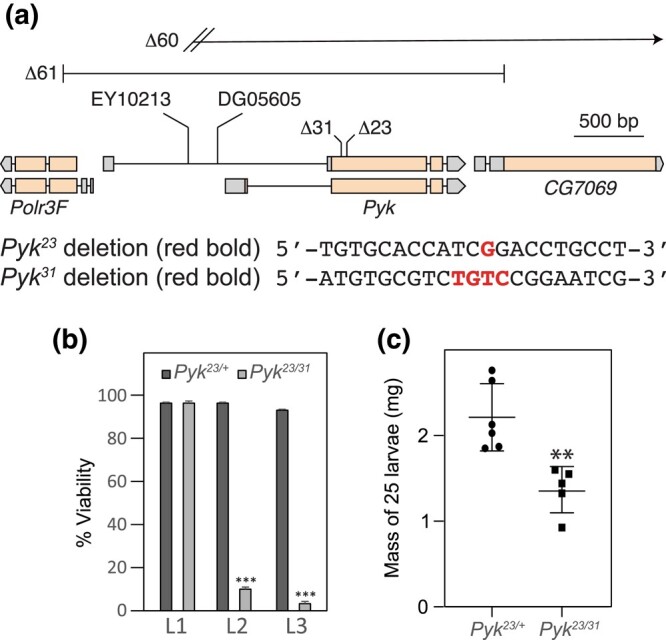
Deletions in the *Pyk*-coding region induce larval lethality and growth defects. a) An illustration of the *Drosophila Pyk* locus and surrounding genes, as well as the *Pyk* mutant alleles. The *Pyk^23^* and *Pyk^31^* alleles were generated using a CRISPR/Cas9-based strategy and induce small deletions that result in frameshifts. The *Pyk^61^* deletion, which was generated through imprecise excision of the p-element insertion *Pyk^EY10213^* (abbreviated EY10213), deletes 6400 bp and removes the entire *Pyk* locus as well as portions of the first-coding exons of the neighboring genes. The *Pyk^60^* deletion was generated through imprecise excision of the p-element insertion *Pyk^DG05605^* (abbreviated DG056050) and represents a large deletion or chromosomal rearrangement of undefined boundaries (denoted by diagonal hatch marks; see *Methods*) that eliminates *Pyk* expression (see Supplementary Fig. 1). Note that *Pol3f* gene expression is normal in trans-heterozygous *Pyk* mutant backgrounds (Supplementary Fig. 1). b) A histogram illustrating the effects of *Pyk* mutations on larval viability. Note that nearly all *Pyk^23/31^* trans-heterozygous mutant animals die during the second larval instar. c) *Pyk^23/31^* mutants are significantly smaller than heterozygous controls. *n* = 6 samples containing 25 mid-L2 larvae. **P < 0.01. ***P < 0.001. *P*-values calculated using a Mann–Whitney test.

**Fig. 3.**
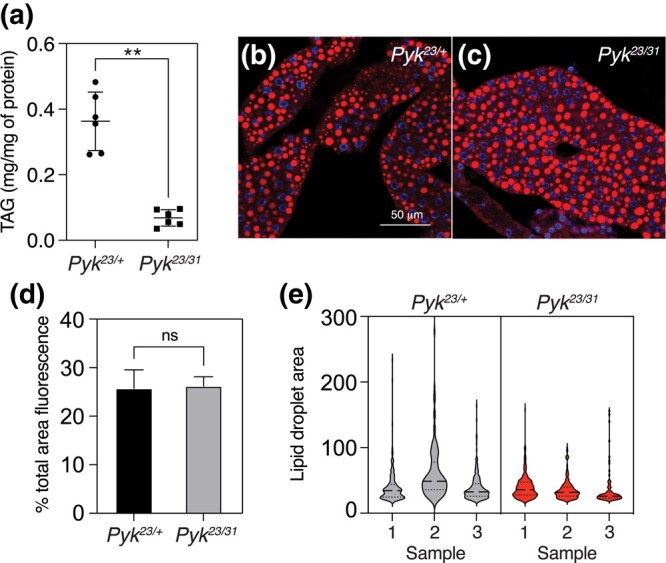
*Pyk* mutants exhibit decreased TAG levels. TAG levels were assayed during L2 development in *Pyk* mutants and heterozygous controls. a) *Pyk^23/31^* mutants exhibit significantly decreased triglycerides (TAG) as compared with *Pyk^23/+^* controls. Data are normalized to soluble protein. *n* = 6 biological replicates containing 25 mid-L2 larvae per sample. **P < 0.01. *P*-values calculated using a Mann–Whitney test. b,c) Nile red was used to stain lipid droplets in L2 fat bodies of both b) *Pyk^23/+^* controls and c) *Pyk^23/31^* mutants. Scale bar in b) applies to c). d,e) Images of Nile red stained *Pyk^23/+^* and *Pyk^23/31^* fat bodies were quantified for d) the percent total area stained by Nile red (% total area fluorescence) and e) lipid droplet size. Three representative images were analyzed for each genotype. Statistical analysis was conducted using a Mann–Whitney test for d) and a Nested t test for e). No significant differences were found in d) or e).

To determine how loss of Pyk activity affects *Drosophila* development, we used CRISPR/Cas9 to generate 2 small deletions in the *Pyk* locus, denoted here as *Pyk^23^* and *Pyk^31^*, both of which induce frameshifts in the first-coding exon and are predicted to be null alleles (Fig. 2a). When these 2 *Pyk* mutant alleles were placed in trans, the resulting *Pyk^23/31^* mutants survived embryogenesis but died during the mid-L2 stage (Fig. 2b). Moreover, when compared with *Pyk^23/+^* heterozygous controls, *Pyk* mutants displayed a significant decrease in both body mass and triglyceride stores (Figs. 2c and 3, a and b), thus demonstrating that loss of Pyk activity significantly disrupts larval development. We would note, however, that although *Pyk* mutants accumulate far less triglycerides (TAG) than controls, the size and density of lipid droplets within the fat body as determined by Nile red staining (Fig. 3, a and b) remain similar to controls (Fig. 3, c and d). Our finding suggests that while loss of Pyk activity results in an overall decrease in TAG stores, the density of lipid droplets with the remaining *Pyk* mutant fat body is maintained at a near normal level. Overall, these findings reveal that *Pyk* mutant larvae display many of the same phenotypes observed in *dERR* and *Pfk* mutants, which also exhibit severe disruptions of glycolytic metabolism (Rust and Collier 1985; Tennessen et al. 2011).

### Metabolomic analysis of *Pyk* mutants

**Fig. 4.**
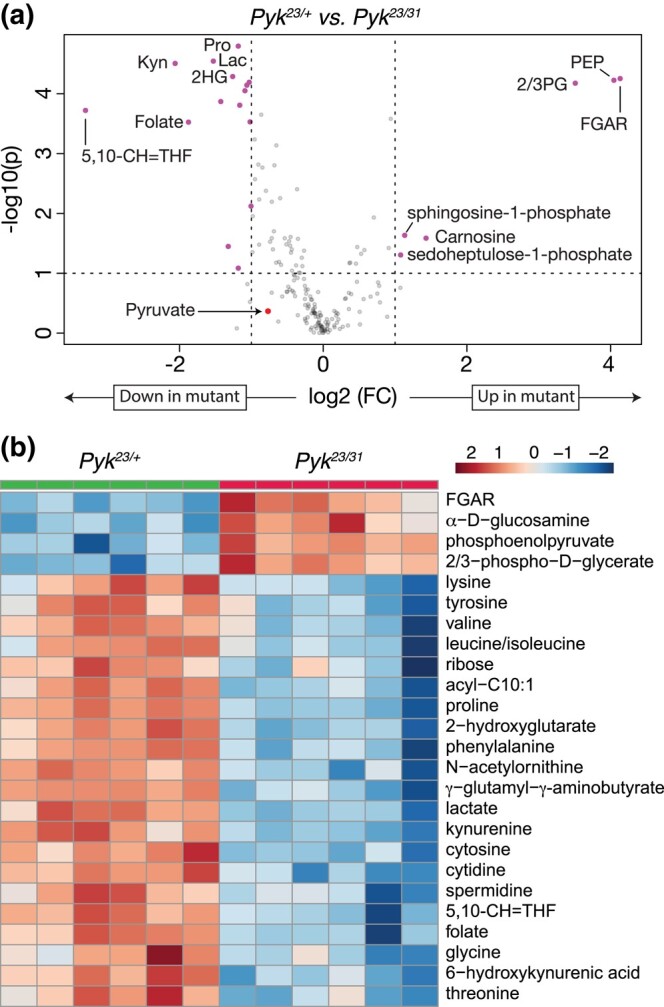
Targeted metabolomic analysis of *Pyk* mutant larvae. *Pyk^23/31^* mutant larvae and *Pyk^23/+^* controls were analyzed using a targeted GC-MS approach. a) Differences in metabolite abundance between control and mutant samples are represented as a volcano plot. Dashed vertical line represents an absolute log2 fold change (FC) of ≥|1.5|. Dashed horizontal line represents *P* < 0.01. Note that pyruvate is not significantly changed in mutants compared to controls. b) A heatmap illustrating the top 25 most significantly altered compounds in *Pyk^23/31^* mutants as compared with *Pyk^23/+^* controls. Both figures were generated using Metaboanalyst 5.0, as described in the *Methods*. Abbreviated compounds are 2/3-phosphoglycerate (2/3PG), phosphoenolpyruvate (PEP), 2-hydroxyglutarate (2HG), 3-hydroxybutyrate (2HB), 5,10-methenyltetrahydrofolate (5,10-CH=THF), alanine (Ala), asparagine (Asn), glutamic acid (Glu), glutamine (Gln), 5′-phosphoribosyl-N-formylglycinamide (FGAR), proline (Pro), and trehalose (Tre). Note that the metabolomics method used to generate this data does not distinguish between 2PG and 3PG.

To better understand how loss of *Pyk* alters larval metabolism, we used a targeted metabolomics approach to compare *Pyk^23/31^* mutants with *Pyk^23/+^* heterozygous controls during the L2 larval stage (Supplementary Table 2). Partial Least Squares Discriminant Analysis of the resulting data revealed that control and mutant samples clustered in distinct groups (Supplementary Fig. 3). Subsequent analysis of the data revealed increased levels of the metabolites immediately upstream of Pyk, 2/3-phosphoglycerate and phosphoenolpyruvate, and a decrease in lactate, and 2-hydroxyglutarate, and most amino acids that can be used as anaplerotic fuel in the citric acid cycle (Fig. 4, a and b, Supplementary Table 2; note that this metabolomics method cannot distinguish between 2-phosphoglycerate and 3-phosphoglycerate. As a result, we refer to the combined measurement of both metabolites as 2/3-phospho-D-glycerate in Fig. 4b). Overall, these changes are indicative of a block in glycolysis at the reaction catalyzed by Pyk.

**Fig. 5.**
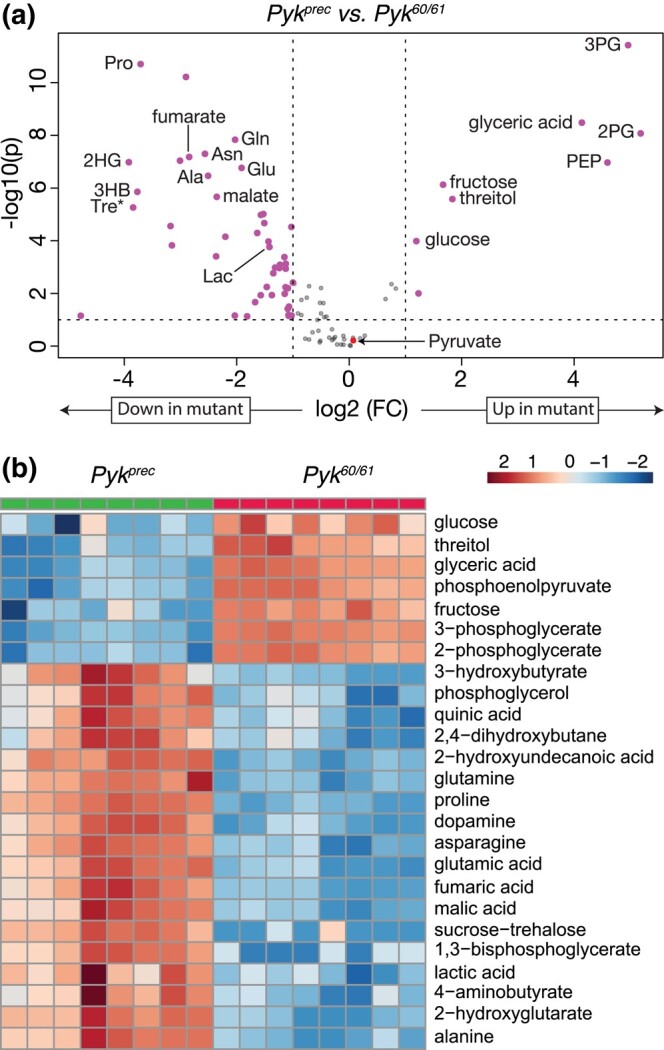
Targeted metabolomic analysis of *Pyk* mutant larvae. *Pyk^60/61^* mutant larvae and precise excision controls (*Pyk^Prec^*) were analyzed using a targeted GC-MS approach. a) Differences in metabolite abundance between control and mutant samples are represented as a volcano plot. Dashed vertical line represents an absolute log2 fold change (FC) of ≥|1.5|. Dashed horizontal line represents *P* < 0.01. Note that pyruvate is not significantly changed in mutants compared to controls. b) A heatmap illustrating the top 25 most significantly altered compounds in *Pyk^60/61^* mutants as compared with *Pyk^Prec^* controls. Both figures were generated using Metaboanalyst 5.0, as described in the *Methods*. Abbreviated compounds are 3-phosphoglycerate (3PG), 2-phosphoglycerate (2PG), phosphoenolpyruvate (PEP), 2-hydroxyglutarate (2HG), 3-hydroxybutyrate (2HB), alanine (Ala), asparagine (Asn), glutamic acid (Glu), glutamine (Gln), 5′-phosphoribosyl-N-formylglycinamide (FGAR), proline (Pro), and trehalose (Tre).

While the metabolite changes observed in our initial metabolomics dataset were largely expected, we were surprised to find that pyruvate levels were unchanged in *Pyk* mutants (Supplementary Table 2). To verify that pyruvate levels remained constant even in the absence of Pyk, we analyzed a second series of *Pyk* mutants using a different methodology in an independent metabolomics core facility. In this case, a trans-heterozygous combination of 2 *Pyk* deletion alleles, *Pyk^60/61^*, were compared with a genetically-matched control strain that was homozygous for a precise excision allele (Supplementary Table 3; see Methods for a description of the alleles used). Although these deletions also removed portions of *CG7069* (Fig. 2a), which encodes another member of the Pyk family, this gene is not expressed during most of larval development (see FlyBase, Chintapalli et al. 2007; Robinson et al. 2013; Brown et al. 2014; Gramates et al. 2022), and thus our analysis likely reflects phenotypes caused by the loss of the *Pyk* locus. Similar to the metabolomic profile of *Pyk^23/31^* mutants, the metabolome of *Pyk^60/61^* mutant L2 larvae differed significantly from the control strain (Supplementary Fig. 4), exhibiting increased levels of glycolytic metabolites immediately upstream of the Pyk-catalyzed reaction [2-phosphoglycerate, 3-phosphoglycerate, and phosphoenolpyruvate (Fig. 5, a and b; Supplementary Table 3)]. We also observed decreased levels of the downstream metabolites lactate and 2HG, as well as several amino acids, in *Pyk^60/61^* mutant larvae compared with the precise excision control (Fig. 5, a and b; Supplementary Table 3). Notably, *Pyk* mutant larval samples also displayed normal pyruvate levels (Fig. 5a), suggesting that the larval pyruvate pool is maintained by compensatory changes in other metabolic pathways.

**Fig. 6.**
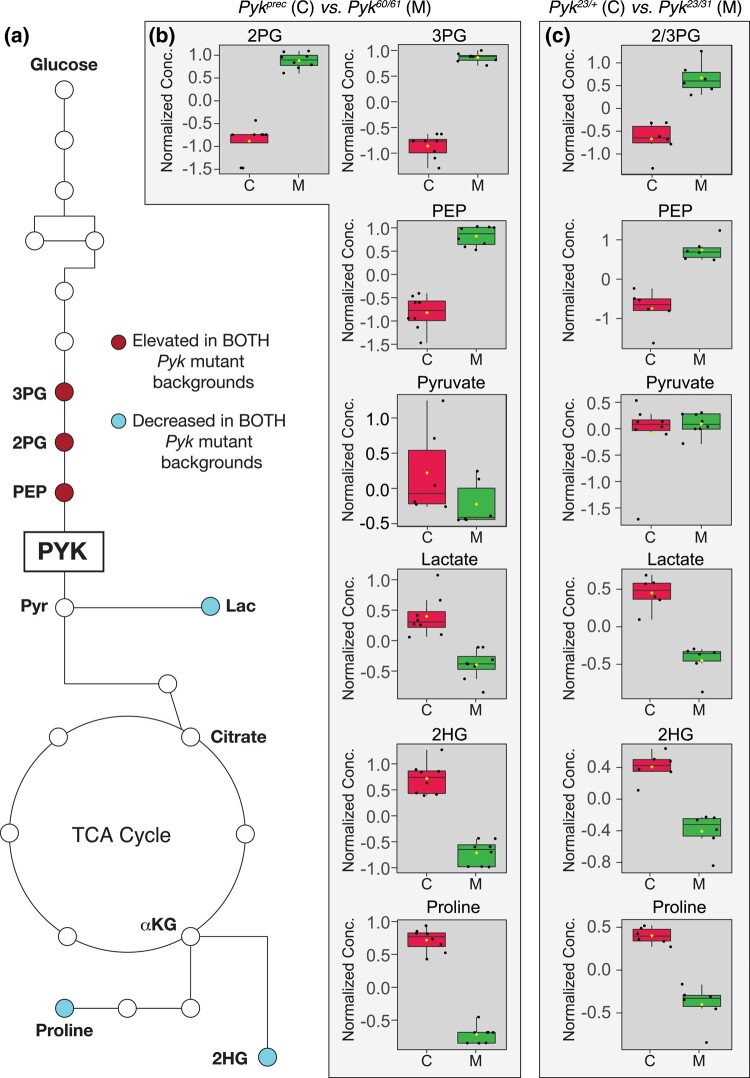
A limited set of metabolites are significantly altered in both *Pyk* mutant backgrounds. a) An illustration of glycolysis and the TCA cycle highlighting metabolites that are significantly altered in both *Pyk* mutant backgrounds. b,c) Boxplots illustrating the change in abundance of metabolites that are significantly altered in both b) *Pyk^60/61^* and c) *Pyk^23/31^* mutant backgrounds. All boxplots were generated using Metaboanalyst 5.0, as described in the *Methods*. Dots represent individual samples, the horizontal bar in the middle represents the median, and diamonds represents the mean concentration. For all boxplots except those for pyruvate, the metabolite fold change was ≥2 fold, *P* ≤ 0.01, and the adjusted *P* ≤ 0.05. Abbreviated compounds are 3-phosphoglycerate (3PG), 2-phosphoglycerate (2PG), phosphoenolpyruvate (PEP), and 2-hydroxyglutarate (2HG). Note that the metabolomics method used to analyze *Pyk^23/31^* mutants and *Pyk^23/+^* controls does not distinguish between 2PG and 3PG.

As an extension of our metabolomic studies, we compared our datasets to identify those metabolites that were similarly altered in both *Pyk* mutant backgrounds. While we would note that this comparative analysis is somewhat limited in that we used 2 different targeted metabolomics protocols that were optimized to detect different subsets of metabolites, molecules that appear in both analyses highlight metabolic pathways that are significantly altered by loss of Pyk activity in different genetic strains. Despite the many metabolic changes observed in each individual dataset, only a handful of metabolites were common among the 2 experiments as follows: 2/3-phosphoglycerate, phosphoenolpyruvate, lactate, 2-hydroxyglutarate, and proline (Fig. 6a–c). While the changes in 2/3-phosphoglycerate, phosphoenolpyruvate, and lactate were to be expected, the decrease in 2HG and proline present an interesting model for how metabolic flux could be rewired in the absence of Pyk activity (see *Discussion*).

### Gene expression analysis of *Pyk* mutants

**Fig. 7.**
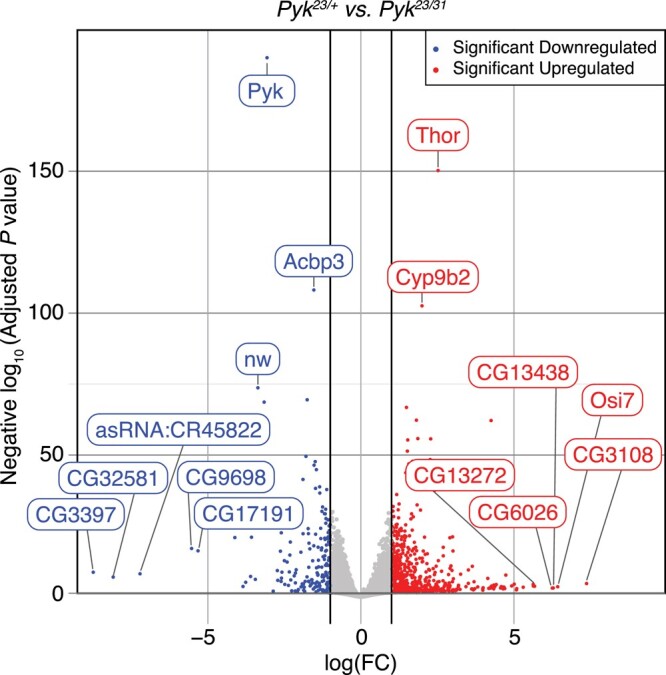
RNA-seq analysis of *Pyk^23/31^* mutants as compared with *Pyk^23/+^* controls. A volcano plot illustrating gene expression changes in *Pyk^23/31^* mutants as compared with *Pyk^23/+^* controls. Black vertical lines indicate absolute log fold change (LFC) ≥ 1. Black horizontal line represents adjusted *P*-value ≤ 0.05. The top 15 most significantly changed genes are labeled.

Our metabolomic studies suggest that loss of Pyk activity induces compensatory changes in other metabolic pathways that allow the larvae to survive early development. To better understand if this metabolic rewiring is recapitulated at the level of gene expression, we used RNA-seq to compare the expression profiles of *Pyk^23/31^* mutant larvae with *Pyk^23/+^* heterozygous controls (Supplementary Table 4). The replicates showed high within-genotype correlation (Supplementary Fig. 5b), with their first principal component corresponding to genotype and nearly 79% of variance (Supplementary Fig. 5a). Differential expression analysis revealed significant changes in gene expression between mutant and control samples, with 755 genes upregulated and 214 genes downregulated more than 2-fold in the *Pyk* mutant dataset vs heterozygote control (adjusted *P*-value ≤ 0.05; Supplementary Table 4, Fig. 7). Notably, the expression of the *Pyk* gene itself was downregulated 8.3-fold when compared with the control strain, likely indicating that the *Pyk* alleles used in this study destabilize Pyk mRNA transcripts, perhaps due to nonsense mediated decay (Supplementary Table 4, Fig. 7).

The RNA-seq data also revealed no significant difference in the expression of the *Pyk* homologs *CG7069* and *CG11249* in mutant samples when compared with controls (Supplementary Table 5), a result consistent with the fact that both genes are primarily expressed in the adult male testis (see FlyBase, Chintapalli et al. 2007; Robinson et al. 2013; Brown et al. 2014; Gramates et al. 2022). Meanwhile, transcripts from the 3 other *Pyk* homologs, *CG2964*, *CG7362*, and *CG12229*, were absent from the processed dataset (Supplementary Table 4). However, reanalysis of the prefiltered RNA-seq data revealed that *CG2964*, *CG7362*, and *CG12229* are expressed at such low levels that the corresponding transcript reads were filtered out of the final dataset; both control and mutant samples contained nearly undetectable levels of their transcripts (Supplementary Table 5). Note that the lack of *CG2964*, *CG7362*, and *CG12229* expression is consistent with previous observations that these 3 genes are expressed at low or very low levels in larvae and are instead primarily expressed in the adult testis (see FlyBase, Chintapalli et al. 2007; Robinson et al. 2013; Brown et al. 2014; Gramates et al. 2022). Together, these findings suggest that loss of Pyk, itself, does not induce compensatory changes in the expression of the other Pyk-like enzymes.

The data also revealed a significant upregulation of the gene *Thor* (FBgn0261560), the fly homolog of 4E-BP (Bernal and Kimbrell 2000), which functions as a metabolic brake that dampens mRNA translation (Hay and Sonenberg 2004; Teleman et al. 2005). The significant upregulation of *Thor* transcript levels hints at 1 possible mechanism by which *Pyk* mutants both rewire larval metabolism and slow growth in response to a major metabolic insult.

To further analyze the *Pyk* mutant RNA-seq data, we used Gene Ontology (GO) to better define the biological processes most affected by loss of Pyk activity. Our analysis revealed that *Pyk* mutant samples exhibited a significant enrichment in GO categories associated with the digestion and metabolism of chitin and glucosamines, lipids, and proteins (Table 1, False Discovery Rate of *q* < 0.1; Supplementary Table 6). Notably, analysis of the RNA-seq dataset using the “Preferred tissue” tool in PANGEA indicated that the digestive system displays the largest number of significantly upregulated genes in *Pyk* mutants (Supplementary Table 7) (Hu et al. 2023), with many of the those genes being involved in amino sugar, lipid, and protein metabolism. Consistent with this finding, most of the significantly upregulated genes that fit within the GO categories “lipid metabolic process” and “proteolysis” encode intestinal lipases and digestive proteases (Table 1 and Supplementary Table 6, Supplementary Fig. 6). Overall, our results suggest that the metabolism of *Pyk* mutants attempts to compensate for its loss by increasing expression of genes involved in the breakdown of macromolecules within the digestive tract, perhaps as a means of increasing the uptake of dietary nutrients.

**Table 1.** GO analysis of genes that are significantly upregulated in Pyk23/31 mutants as compared to controls.

GO term	Description	*P*-value	FDR *q*-value	Enrichment (*B*,*b*)
GO:0042335	Cuticle development	1.94^−12^	1.46^−08^	3.62 (168, 38)
GO:0040003	Chitin-based cuticle development	4.06^−11^	1.53^−07^	3.5 (160, 35)
GO:0006030	Chitin metabolic process	2.80^−07^	7.03^−04^	3.41 (103, 22)
GO:0006022	Aminoglycan metabolic process	5.61^−07^	0.0011	3.09 (124, 24)
GO:1901071	Glucosamine-containing compound metabolic process	1.09^−06^	0.0016	3.17 (111, 22)
GO:0006629	Lipid metabolic process	1.51^−06^	0.0019	1.99 (410,51)
GO:0006040	Amino sugar metabolic process	1.75^−06^	0.0019	3.08 (114, 22)
GO:0035337	Fatty-acyl-CoA metabolic process	3.25^−05^	0.031	5.14 (28, 9)
GO:0021556	Central nervous system formation	9.65^−05^	0.080	8.88 (9, 5)
GO:0035336	Long-chain fatty-acyl-CoA metabolic process	1.14^−04^	0.086	6.85 (14, 6)
GO:0006508	Proteolysis	1.20^−04^	0.082	1.59 (622, 64)
GO:1901568	Fatty acid derivative metabolic process	1.37^−04^	0.086	4.36 (33, 9)
GO:0008202	Steroid metabolic process	1.63^−04^	0.094	3.9 (41, 10)
GO:0016042	Lipid catabolic process	1.78^−04^	0.096	2.66 (102, 17)

Analysis was conducted using Gene Ontology enRIchment anaLysis and visuaLizAtion tool (GOrilla). The enrichment value is defined as (*b*/*n*)/(*B*/*N*), where *N* is total number of genes, *B* is the total number of genes associated with a specific GO term, *n* is the number of genes significantly upregulated in Pyk mutant larvae, and *b* is the overlap between genes in the GO category and the upregulated genes in Pyk mutant larvae. For this analysis, *N* = 9,158 and *n* = 573. See Supplementary Table 5 for the genes associated with the enrichment term *b*.

**Fig. 8.**
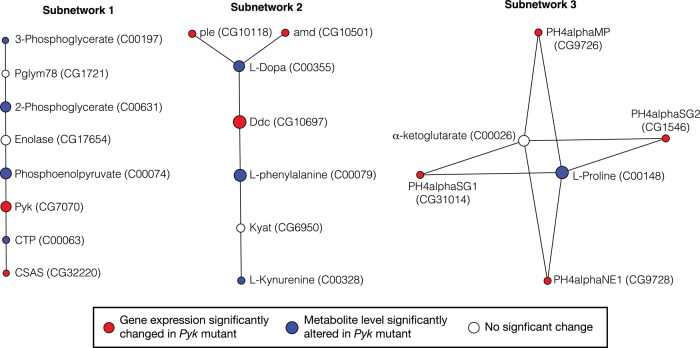
Pathway enrichment analysis of metabolites and genes significantly altered in *Pyk^23/31^* mutants compared with *Pyk^23/+^* controls. Those metabolites and genes that exhibited significant changes in *Pyk^23/31^* mutants compared with *Pyk^23/+^* controls were analyzed using OmicsNet 2.0 for pathway enrichment. Illustrated are the 3 metabolic subnetworks that contained significant changes in both gene expression and metabolite abundance. Values in parentheses correspond to either the gene name listed in Flybase or the KEGG ID for metabolites.

Finally, we used OmicsNet 2.0 to analyze both the RNA-seq and metabolomics data for metabolic subnetworks that are disrupted in *Pyk^23/31^* mutants (Zhou et al. 2022). Our analysis identified 3 subnetworks containing both transcripts and metabolites that were significantly altered in *Pyk^23/31^* mutants when compared to in *Pyk^23/+^* controls (Fig. 8). In this regard, the first gene-metabolite subnetwork identified by our analysis consisted of the last 3 enzymatic steps in glycolysis, the nucleotide CTP, and the enzyme CMP-sialic acid synthase (Fig. 8, subnetwork 1). The relevance of this subnetwork makes sense in that *Pyk* mutants exhibit decreased Pyk transcript levels while also accumulating upstream metabolites. The other 2 subnetworks represent a series of reactions from phenylalanine metabolism (Fig. 8, subnetwork 2), as well as an involving proline and 3 enzymes that normally hydroxylate proline residues within polypeptide chains (Fig. 8, subnetwork 3). These observations suggest that loss of Pyk activity influences processes related to the metabolism of 2 key amino acids.

## Discussion

Here, we demonstrate that *Pyk* mutants exhibit a larval lethal phenotype with defects in larval growth and a severe block in glycolysis. While the phenotypic consequences of mutating *Pyk* are largely expected, our study contains several findings that are important for both the *Drosophila* and biomedical communities. First, we demonstrate that while the *Drosophila* genome encodes several pyruvate kinase paralogs, the gene *Pyk* encodes the canonical enzyme most analogous to Pyk in other organisms. Future studies should more closely examine these paralogous enzymes and determine why *D. melanogaster* evolution produced such a specialized set of pyruvate kinases.

A second key finding from our phylogenetic analysis is that *Drosophila* Pyk exhibits nearly equivalent sequence similarity to both the mammalian PKLR and PKM orthologs. This observation is important because PLKR and PKM are associated with distinct disease phenotypes. Mutations that disrupt *Pklr* result in the congenital disorder PKD, which is manifest in a diverse array of symptoms, ranging from anemia and neonatal hyperbilirubinemia to iron overload, cirrhosis, and endocrine dysfunction (Grace et al. 2018). In contrast, the role of PKM in human disease is mostly studied in regard to the enzymatic differences between the 2 isoenzymes PKM1 and PKM2. Since PKM2 is less efficient than PKM1, altering the ratio of PKM1:PKM2 in a cell can alter the rate of pyruvate production and fundamentally change cellular metabolism (Ye et al. 2012; Israelsen and Vander Heiden 2015). As a result, inappropriate elevation of PKM2 is associated with onset of the Warburg effect as well as hypertension and Alzheimer's disease (Caruso et al. 2017; Zhang, Wang et al. 2017; Hsu and Hung 2018; Traxler et al. 2022). Together, these human phenotypes highlight the specialized role of Pyk isoforms in mammals and demonstrate a need for studying PKM and PKLR in vivo. Our study suggests that careful consideration must be taken in using the endogenous *Drosophila* Pyk gene as a surrogate for studying the unique mammalian roles of PKLR, PKM1, or PKM2.

While future studies in the fly should be cautious when making comparisons with disease states that are unique to either of the human enzymes, the *Pyk* mutants described herein can serve as tools understand how *Drosophila* cells and tissues respond to a general changes in Pyk activity, similar to previous studies that manipulated *Drosophila* Pyk activity in imaginal disc development and neurons/glia (Tixier et al. 2013; Volkenhoff et al. 2015; Wu et al. 2018; Spannl et al. 2020). Moreover, 1 exciting possibility would be to use our newly described mutants as a genetic tool for expressing disease-causing Pyk variants. After all, pyruvate kinase deficiency is the most common enzymatic disorder detected in newborns, and a wide variety of disease-causing PKLR variants have been identified (Dolan et al. 2002; Grace et al. 2015; Fattizzo et al. 2022; Luke et al. 2023). Expression of the wild-type and mutated human enzyme variants in the fly would allow for a rapid genetic analysis of how PKLR mutations alter cellular metabolism while also providing an in vivo platform to test potential therapeutics.

We would also highlight that, based on the phylogenetic analysis conducted here, the *Pyk* gene in *D. melanogaster* is most homologous to the previously described *A. aegypti* protein PK1 (Petchampai et al. 2019). The high degree of homology between *Drosophila* and *A. aegypti* Pyk enzymes suggests that activity of the fly enzyme could be regulated in a manner similar to that of the mosquito. Notably, *A. aegypti* PK1 is allosterically activated by a unique set of amino acids (alanine, glutamine, proline, and serine) when compared with other animals (Petchampai et al. 2019). While similar studies have yet to be conducted in the fly, we would note that *Drosophila* Pyk activity is likely specialized for the unique ecological niches occupied by this animal. In this regard, *Drosophila* Pyk physically interacts with the oxygen-sensing enzyme Fatiga (Chen et al. 2011), a prolyl hydroxylase that regulates stability of the *Drosophila* transcription factor sima, a homolog of the HIF transcription factor (Gorr et al. 2006). Considering that *Drosophila* larvae require aerobic conditions for normal growth but occupy hypoxic niches, the putative interaction between Fatiga and Pyk warrants further investigation (Callier and Nijhout 2014).

Our studies of the *Pyk* mutant also reveal how a block in glycolysis alters systemic metabolism in unexpected ways. Previous studies in the fly have examined how disruption of glycolytic metabolism affects both the metabolome and transcriptome. For example, the *Drosophila* Estrogen-Related Receptor (dERR) is a central regulator of glycolytic metabolism throughout the fly lifecycle (Tennessen et al. 2011; Beebe et al. 2020). Our studies reveal that the developmental defects exhibited by *Pyk* mutants largely phenocopy those observed in *dERR* mutants although the *Pyk* and *dERR* mutant metabolomic and transcriptomic profiles differ significantly. Unlike *dERR* mutants, which display a buildup of sugars due to the downregulated expression of nearly every glycolytic enzyme (Tennessen et al. 2011; Beebe et al. 2020), *Pyk* mutants instead accumulate 3 glycolytic metabolites immediately upstream of the Pyk-catalyzed reaction. Thus, our studies of *Pyk* highlight how the *dERR* mutant metabolic profile represents a broad disruption of carbohydrate metabolism when compared with the loss of a single *dERR* target gene.

While most aspects of the *Pyk* mutant metabolomic profile were expected based on the known role of Pyk, several interesting observations emerged. First, levels of pyruvate were unchanged in *Pyk* mutants as compared with controls. Since the other metabolites in glycolysis are changed in a manner that is indicative of a loss of Pyk activity, and both lactate and TCA cycle intermediates exhibit a severe decrease, flux into and out of the larval pyruvate pool must be severely compromised. Future studies should explore this possibility using stable-isotope tracers and further examine how larval metabolism maintains pyruvate levels even under severe metabolic stress.

Our analysis also raises questions about how *Pyk* mutants, as well as *dERR* mutants, survive until the mid-L2 stage. Glycolysis is severely disrupted in both mutant backgrounds, yet these animals continue to develop for almost 3 days when raised under standard culture conditions. While this impressive resilience might be partially explained by maternal loading of Pyk and other enzymes, both metabolomics and transcriptomics analysis suggest that *Pyk* mutants switch fuel sources, possibly in an attempt to maintain developmental progress. Notably, *Pyk* mutants exhibit a significant decrease in the fermentation products lactate and 2HG, as well as a decrease in the amino acid proline. All 3 changes signal a major reorganization of larval metabolism, as lactate and L-2HG are present at high levels within normal larvae and represent pools of carbon that are not immediately used for energy production and growth (Li et al. 2017). Meanwhile, proline is commonly used for energy production in insects (Teulier et al. 2016).

Thus the depletion of lactate, 2HG, and proline pools suggests that *Pyk* mutant metabolism has become reliant on alternative energy sources, such as proline, and lacks adequate resources to ferment pyruvate and a-ketoglutarate into lactate and L-2HG, respectively. This hypothesis is also supported by RNA-seq analysis, which uncovered a significant upregulation of enzymes involved in the digestion and metabolism of complex lipids and proteins. Overall, further studies of *Pyk* mutants are warranted to understand how these metabolic adaptations are induced and regulated.

Our results also notably revealed that *Pyk* mutants display developmental phenotypes that differ from a previous study of *Mpc1* mutants, which are unable to properly transport pyruvate into the mitochondria (Bricker et al. 2012). Considering that Pyk and Mpc1 both directly affect pyruvate metabolism, one might predict that mutations in these genes would produce similar phenotypes. Instead, *Mpc1* mutants are viable when raised on standard *Drosophila* media and only exhibit a lethal phenotype when placed on a sugar-only diet (Bricker et al. 2012). Moreover, the metabolomic profile of *Mpc1* and *Pyk* mutants display important differences. Specifically, the earlier *Mpc1* study revealed that serine levels are significantly increased in *Mpc1* mutants (Bricker et al. 2012)—an observation that would be consistent with an uncoupling of glycolysis and the TCA cycle causing a rerouting of glycolytic intermediates into the serine/glycine biosynthetic pathway. In contrast, we find that *Pyk* mutants exhibit no increase in serine production despite harboring very high levels of 3-phosphoglycerate, which serves as the precursor for serine biosynthesis (Ye et al. 2012). This difference is surprising because studies of PKM2 in cancer cells suggest that decreased Pyk activity induces shunting of glycolytic intermediates into serine production (Ye et al. 2012).

One explanation for the dramatic phenotypic differences between *Pyk* and *Mpc1* mutants stems from differences in lactate metabolism. Unlike *Pyk* mutants, *Mpc1* mutants accumulate excess lactate (Bricker et al. 2012). Thus, *Mpc1* mutants can generate 2 more ATPs from each glucose molecule when compared with *Pyk* mutants, allowing for regeneration of ATP independent of mitochondrial activity. Future studies should test this hypothesis by examining if *Mpc1;Ldh* double mutant exhibits metabolic and developmental phenotypes that more closely mimic those observed in *Pyk* mutants.

In conclusion, we have here described a metabolomic profile of *Drosophila* larvae lacking Pyk activity. While the resulting changes in central carbon metabolism are largely expected, such an analysis of fly metabolism is essential as *Drosophila* geneticists increasingly study the role of metabolism across a wide range of contexts. Moreover, *Drosophila* metabolism is highly adaptable and compensates for metabolic insult—as seen here by depletion of the anaplerotic amino acid proline. A careful exploration of these metabolic networks will be essential for studying metabolism in the context of *Drosophila* growth, development, and models of human disease.

## Supplementary Material

jkad228_Supplementary_Data

## Data Availability

All strains and reagents are available upon request. All targeted metabolomics data described herein are included in Supplementary Tables 2 and 3. Processed RNA-seq data are presented in Supplementary Table 4 and available in NCBI Gene Expression Omnibus (GEO; GSE234299; Heidarian et al. 2023). Supplemental material available at G3 online.
