# Supplementary material for: Metabolomic analysis of Drosophila melanogaster larvae lacking pyruvate kinase
Source: G3 (Bethesda). 2023 Oct 4;14(1):jkad228. doi: 10.1093/g3journal/jkad228 (PMC10755183; doi:10.1093/g3journal/jkad228)
Supplement: jkad228_Supplementary_Data [file jkad228_supplementary_data.zip › Figure_S1_G3-2023-404572.pdf]

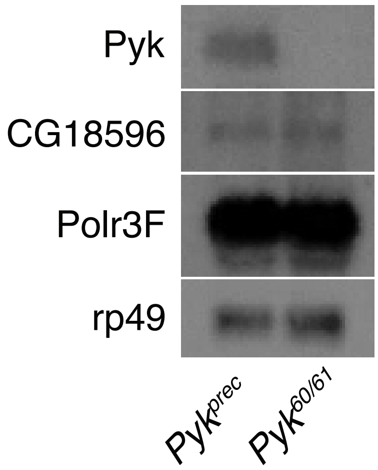

Figure S1. *Pyk* mRNA transcript levels are significantly reduced in *Pyk* mutant larvae. Total RNA from stage w^{1118}; Pyk^{prec} control larvae and w^{1118}; $Pyk^{60/61}$ mutant larvae were analyzed by northern blot hybridization to detect transcripts encoding Pyk, CG18596, and Polr3F. Hybridization to detect *rp49* mRNA is included as a loading control.
