# Supplementary material for: Metabolomic analysis of Drosophila melanogaster larvae lacking pyruvate kinase
Source: G3 (Bethesda). 2023 Oct 4;14(1):jkad228. doi: 10.1093/g3journal/jkad228 (PMC10755183; doi:10.1093/g3journal/jkad228)
Supplement: jkad228_Supplementary_Data [file jkad228_supplementary_data.zip › Figure_S2_G3-2023-404572.pdf]

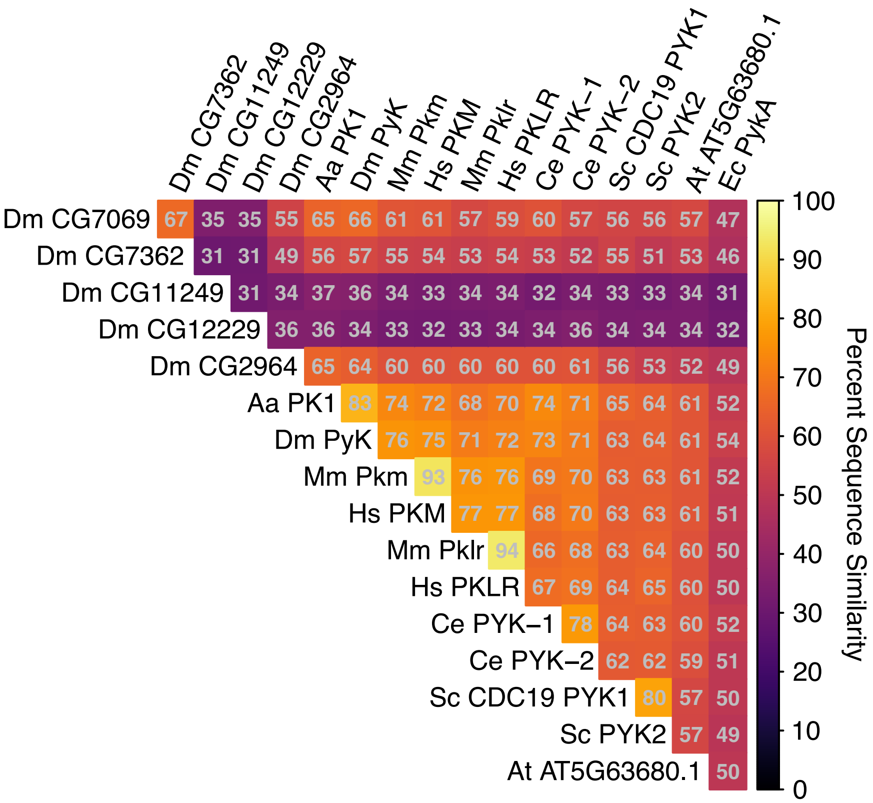

Figure S2. A comparison of sequence similarities between Pyk homologs. A heatmap of pairwise sequence identities extracted from the ensemble MSA. Species abbreviations: Aa, A. aegypti; Dm, D. melanogaster; Hs, H. sapiens; Mm, M. musculus; Ce, C. elegans; Dr, D. rerio, Dd, D. discoideum. See Supplemental Table 1 for a list of isoforms used in this analysis.
