# Supplementary material for: Metabolomic analysis of Drosophila melanogaster larvae lacking pyruvate kinase
Source: G3 (Bethesda). 2023 Oct 4;14(1):jkad228. doi: 10.1093/g3journal/jkad228 (PMC10755183; doi:10.1093/g3journal/jkad228)
Supplement: jkad228_Supplementary_Data [file jkad228_supplementary_data.zip › Figure_S4_G3-2023-404572.pdf]

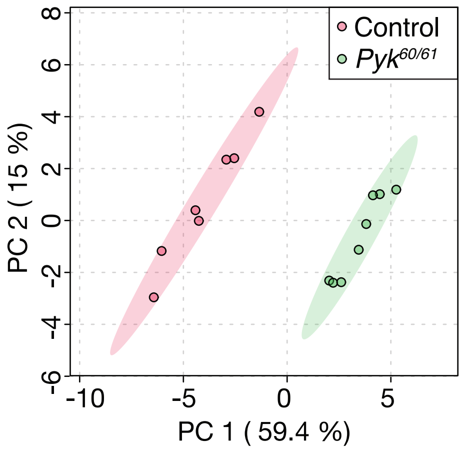

Figure S4. A comparison of the metabolomic data from *Pyk*$^{60/61}$ mutant and *Pyk*^{prec} control samples using Partial Least Squares Discriminant Analysis (PLS-DA). Targeted metabolomics data from Table S3 was analyzed using PLS-DA. Analysis was conducted using Metaboanalyst 5.0.
