# Supplementary material for: Metabolomic analysis of Drosophila melanogaster larvae lacking pyruvate kinase
Source: G3 (Bethesda). 2023 Oct 4;14(1):jkad228. doi: 10.1093/g3journal/jkad228 (PMC10755183; doi:10.1093/g3journal/jkad228)
Supplement: jkad228_Supplementary_Data [file jkad228_supplementary_data.zip › Figure_S6_G3-2023-404572.pdf]

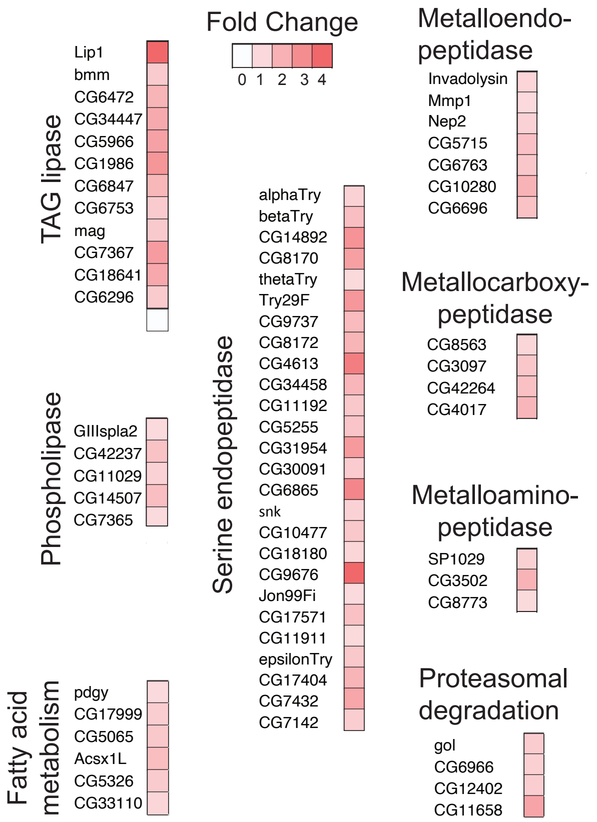

Figure S6. Expression of intestinal lipases and proteases are upregulated in *Pyk* mutants. Heatmaps depicting the expression of significantly up-regulated genes in the GO categories “lipid metabolic process” and “proteolysis”. These genes are grouped by enzymatic function, with the increased fold-change in gene expression represented by a color gradient (log2-fold change in $Pyk^{23/31}$ mutants compared to $Pyk^{23/+}$ controls).
