# Supplementary material for: Metabolomic analysis of Drosophila melanogaster larvae lacking pyruvate kinase
Source: G3 (Bethesda). 2023 Oct 4;14(1):jkad228. doi: 10.1093/g3journal/jkad228 (PMC10755183; doi:10.1093/g3journal/jkad228)
Supplement: jkad228_Supplementary_Data [file jkad228_supplementary_data.zip › File_S1_G3-2023-404572.docx]

**File S1. Sequence of the *Pyk^61^* deletion**

>3R:22366870-22373269(+)

GATGCCTTTGTTGCCGCCCTCCTCAACAATGCCGTAGACGACCTTCTCCTCATTGTCCGCATCCTTGGGCAGGGCACTCT

TCTTTTCCGGGTCCTTGGCACGGTACACCAGCTTTTCACCCTTCTTCAGGATCTCGATCCCGCCCTCCTGCAGCAGTATA

TTCAAAGCTTCCACCCGCGTGGCCGCCGGCACATCGGGCAGCGCCTTGGTCAGGTCATCGTTGGTGGCACCGGCTGGGAT

GCCCTGGACCACCGCCAGCAAAAGTTGCGACACCTCAGTGGCCATTTTTCTATTCTCGAAAATTTGTTATTTAAAATTCA

ATTAAATCGAGAAAAACAACACGCTTACCGTGAAAAGCAAAACAAAAACAAACGGTTCCGGGCGAATGGCTACCGCTGCT

AAGAAGAAGCTAAAAAATAACAAAAATCACAAACCAATTTTAAAAATATAAGTTTGTTCTATTAAAAATAATACTTTACG

CAACTGAATAAATGAGGATAAATAAGGAGCATATGTTTTAGGCTTTATTACAAATTGCTTTAAATAAAATTTAAAGCTTT

CCGAAGACCCCAAATGTTATATCGTGAATCTGGTATGTATATACCAAATGGTATTTATTTTAAAAACGAGTGGCTTTTTT

AGGCTCATTGGTGCCAACTGTTTTTTCGAACAGATCGCCGGACGCACTGTTTCTGATTGGAGTGTTCGCTGCCTGTGCAT

TTAAATTGAATTAAAATAATTCCAAAACAGGAAGAGCAAATACATCTTTCCGCGAGTTCACCTTTACTGTCAAAAACAGG

TAATTATCAGATCTTCTTGATACGACCTCAAGCTAAACATCATTCCATTATAATACGCTAGAAATTTTGCTAACGAAATT

GGCGGGGGCAAGACATTTGTTTTTGCTGTTGTTGATGATGCAAGCAATTTCTCCACATTATGTAGAATTGCGTTTGCCGC

CTCACAAAACACATACACACAAGCACGGCTGCGAATTCAATTCGCACACCCATACAATCTCAAAAGAACCTCGTTAGCGA

AATGTGTAAAGGTGAAATAGAAGTCATTCCATGCGGAGCGCGCCACAAGTAAAATAACCTACACCATCGCAATCACACCC

TATTAAACATGCCATCGGAAATAATGCAGCAATAATAATCTTGAACCGCTGTAAACTTCCACTATCGCAATTGTTTAATT

GCTCTGTGACGACGACGCTTTCAGCGATGATGAGTAATTTTGCATAAATTTGCTCTGTGCCGGATTGTCTGAATTATATT

TGAAATTTTGTTGCTGTTTCGGCGTCTCAGTGATAAGCATTTGTTGTTGCAGACCCGAGCTTCAAAATGTTAACGGCGCT

AACGTCCTTTATCCACAAGTTCAAGGTTAAATAGTACGGCACATAGTACTCATTACATATTGACTGAAGCCTGGAAAAGC

ACTTTCACTTTCAGAACTTTTCTCGTGCGTTTTCCGGCGACAAAAGCTGCAGGCCGGCGAGCTTTACTTGCACGGTTTCT

GCGCGTGTCACTCACGATGAGCGAAAGAGAGAGCGGAAGATAAAAGGAAAGTAGATGCTCACGCACTTGCATAATGGCAG

TGCGAGCGAGACGAAGCTGTTTTCTGTGGAGGAGATTAGGTTGTCAAGGTCAGCACAGTGGAATTCTAGGTGGAAGTGGG

ACAGTTGGACAGTGGGCTCTTTTCCGCGTTGCTTTTTTTCTGTATTGCCAGCTGATAAGCGCCGTCTAACAGCTGATGAT

CGGTTTTTTGTTTACCACGCACACACATACTGACATCACGTGTCCATATAATCATATATACACATACTTCTGTACAATGC

AAATTTATATGCAAAAATTAATAATCGTAATGGATCGTTGAACCTTCTGTTCTATCCGATTGCCGGCCAGCCTAGCAACG

GAGCTTTGCGCCAGTTAACTTCGGAGCTTCCTTTTTGGATAAACTTTTCGCATCCCCTTACGCGAAGGGAGTTAGGGTAT

GTTGGCACAATAGCATATCTTGATCCTGCTAAACACTTTACTTATACTTAAATATCGAATGATAATTTGAGCCTCTATCT

TTTCCATTTGATAATATCTTTATTAATTTTTATATGCAGTATTGTAAGATTAAGGTTATATTTCACTCGGAATTATATTG

GGAATAGCGGTAAGGCCACTTGGTCGACTGAGCAGCTGGCAGTCCCTGGTCGAGCATACCCTTGAATATACCCTTCTTGC

TCGCTCACCAATGTTGTCTCTTTAGGGGTGGCCTGTTGCGTCCGCCTCTTGCCTCTTGGGCCCAAACTCTCTCTCCTCTG

CGTTTATGCTCTCTTTGCTGTCGGGCTACCGACATTCTGAGCGCTTTTTCTGCGGCTAGGAAAATTTCCGCTTGCCAAAA

AATCTGTGTGGCAGTCGAACTTTTTGTCTCGTCCTCGCCGCGTCCGATTTTCCATATATCCCGATTTTCGTCTCAAACTT

GGAAGATATATTGCCAAAATATTGCATTCGGCTAGCCGCTGCGTTGTGTGTGTTGTTGTGCGTGGAGTTGCGTGTGCAGT

TGCAAGAAGATATAGTCGTCTATATCGTCAGACCGATCTCGATCCCAAGTGCGATTCCCGTTGGTTACATACTACCCGTT

CGGTAGGCAACAAAATGGTGAACGTAACCATCTACGATGAGGCACCGCAGCTGAAGGTAGGCCAATGTCACGGTAATGGA

AGCCCCCAGAGTTGGTCCATAAAGATATTCATAATCGCCATAATCAGTTGGCTGGCGAGTTGGCCCGATATCAGAGTCAT

TGGGGAGGAAAATGTGGGAAAAGACAATTATGCAACTGCAGCTGAGAAAATTCTGTCGGTCGCGGTCGGCAGAACTGACC

TTCGGCGGTGCAGATGTGCAGCGCTGCCGGTGTCGGCTTCGCTGCACACTTTTCTTCCGCCTGCTGCTTTCTCTCTGCCA

CTCTCTCTTCCGCTCTGCCTTTCTCAGGTCCTCTCTACGGGGGACACCCCTAACCACCCACGCCCATCCACTCAAGCACT

TCAAGTACACGCACTTTGTTTACATCAGCGAATTCTGCAGCGACAGCTTTGCGTTTTTCAAAACATAACATGCACAGTGC

GAATAAATATTTTGATAATTTAAGCAATCGTAAGAGTTAACAAATAGTATTTAACGCAACAGCATCATTTAATATTGGTA

ATCGATTATAACATTTTATATGTCAAGTTTTATCAATGGCTTATTTACACATGCTACTAAGTACCATCAGAATGATAAAA

TACTGTTAATATATAACGTTATCATTTTAATTAGGATTATTAGATTATTTAAAATCATTACCTTTATATGTAAATAGCTC

TATTTTATCTGTCAGTTCACAAATATTTCAATCATTCCTTTTGGTCATGAAAAATGATTGGTGCGGCGAGCATTTGGGCT

TAAATCGCCGTATTTGGCCCGTTTGGTTCTATTTATAAGCCGGTGGTAAACATTTAATTTGGTCGATAAAAACTGTATCG

ATTTATATTCGTTGATATGAAGAAGGGTTATCATGAGTTTTGCACGCATTCGCCACGTTTATGTTGGGGTCCTAAATGTG

TTTCGCATTGCATGACTTCACCAGGGCATGATTTTAATATCATAAAACTAGTTGGTTTTTTTATTTTTTGCTAGAACTTG

GTAGTTTTTAAAACGCGTAGGGTTTCTTGTTGCTAGGAAGAGACTTCTGATTATTAGCGTAAATTGGAAATAAATGCTTA

AAATCAATAAACACGACTTTAACCTGTTTTATTGCAAAAGTTGGCAATCAGCGTGTTGATTTCTAAAAATAAAGAATCAA

TTCGCGCTGTTTATAAATATGAAGAGAAATGAAAACAAACAGAATTAAGTATAGTTTTCATTTTGCAGCCCAACGAAGTA

CCCCAAAACATGGCCGCAGGAGCTGATACCCAACTGGAGCACATGTGCCGTCTGCAGTTCGACTCGCCAGTGCCCCATGT

GCGTCTGTCCGGAATCGTGTGCACCATCGGACCTGCCTCCAGCAGCGTGGAGATGCTGGAGAAGATGATGGCCACCGGCA

TGAACATCGCGCGCATGAACTTCTCTCACGGATCGCACGAGTACCATGCCGCCACCGTGGCCAATGTGCGCCAGGCGGTG

AAGAACTACTCGGCCAAGCTGGGCTACGAACACCCCGTGGCCATTGCCCTGGACACCAAGGGGCCCGAGATCCGTACCGG

TCTGATCGGAGGCAGCGGCACCGCCGAGATTGAGCTGAAGAAGGGCGAGAAGATCAAGCTGACCACCAACAAGGAATTCC

TGGAGAAGGGCTCTCTGGAGATTGTGTACGTGGACTACGAGAACATTGTCAATGTGGTGAAGCCCGGCAACCGGGTGTTC

GTCGATGACGGTCTGATCTCACTGATTGTCCGCGAGGTAGGCAAGGATTCCCTCACCTGCGAGGTGGAGAACGGCGGCTC

TCTGGGTTCCCGCAAGGGTGTGAACCTGCCAGGCGTGCCCGTCGATCTGCCTGCCGTCTCCGAGAAGGATAAGAGCGATC

TGCTGTTCGGTGTGGAGCAGGAAGTGGACATGATCTTTGCTTCGTTCATCCGCAACGCCGCTGCTTTGACCGAGATCCGT

AAGGTTCTTGGCGAGAAGGGCAAGAACATCAAGATCATTTCCAAGATCGAGAACCAGCAGGGCATGCACAACCTGGACGA

GATCATCGAGGCCGGTGATGGCATTATGGTGGCCCGTGGAGATCTGGGTATTGAGATTCCCGCCGAGAAGGTGTTCCTCG

CCCAGAAGGCCATGATTGCCCGCTGCAACAAGGCTGGCAAGCCTGTGATCTGCGCCACTCAGATGTTGGAGTCAATGGTG

AAGAAGCCACGTCCCACTCGCGCTGAGATCTCTGATGTGGCCAACGCTGTGCTCGATGGTGCTGATTGCGTCATGTTGTC

TGGTGAGACCGCCAAGGGCGAGTACCCGCTGGAGTGCGTCCTGACCATGGCCAAGACCTGCAAGGAGGCCGAGGCTGCCC

TCTGGCACCAGAACCTCTTTAACGACTTGGTTCGCGGCGCTGGTACCATCGATGCCTCTCACGCGGCTGCCATCGCTGCC

GTTGAGGCTGCCACCAAGGCCAAGGCCTCCGCCATCGTGGTGATCACCACCAGCGGCAAGTCGGCCTTCCAGGTGAGCAA

GTACCGCCCACGCTGCCCCATCATCGCGGTCACCCGTTTCGCGCAGACCGCCCGACAGGCCCATCTCTACCGTGGACTGG

TGCCACTCATCTACAAGGGTAGGTTTTTGTTTCTCCAGGTCGTTCTTTAAATTATAACTCTGGCTTGTACTTGCAGAGCC

CGGTCTTGGTGACTGGCTGAAGGACGTGGACGTGCGCGTGCAGTTCGGTCTGCAGGTCGGAAAGAAGAACGGCTTCATCA

AGACCGGCGATTCCGTCGTGGTGGTTACCGGCTGGAAGCAGGGCTCCGGCTTCACCAACACCATCCGCATTGTGTAAGTT

TAAATTCTAGCATGTAGATATATATGTATCTGTGCAAAAATTATATCAACAAAACTAACGCAATTATCTATCATTTCAGC

ACCGTCGAATAAATTCTGCCGCTCAGGAAGTACCTGGATGCGTCGCAGCTGCCGCGGGCGTCGCGTTGATTACTGTTAAT

AACCAAGTTAATTTATTAATTACACAATATTCGGAATATTGGAAACTGCAGGAGGCTGCTGGAACCGCTGTCAGAAGCGC

CGAAGGCCGGCCCGGGCCTCAAACAGACACACAAATATACTATACCATTGTTGAATAATAGAGATTTCTTCGAAAATTAT

AATAAAGTCATCACTGTATTTATGTACGTATGGAAAGTAATGTGCCATTTTCTCCTTATACTTGGAACCTTTGGGTATTA

GGTCTGCATGTACGAGATCCACATCCAGTTCCCTATAGGCAATTGATTTTATTCGTTTTTGACAGGTAACACTACATTTT

ATTTTTGTTTCGGTGTATCAAAATTTATTCAAAATTCATCCATACGCAAGAATCCTTAGTCCAGGGTCCACAGTGCCCAT

ATGTTCCACTTGCCTGTTAAACCTAAGATATCGCTGCTATGCCAGCAAATGCAAGAAAGAGAAGGTAGGACGTTGTTACG

TTGGGAGTCAATCATCCGCTTATGAATTCTTTCCAGAAACCACCGAAGTTCCGACTGGACCGCGGACCATTCGCCTCCCA

GTTGGATTACCAGTCGCGACTGCAGTACCAAGCACCTGCCCTGAGTATCCCCCTCAGCAGCATCATATGCACCATTGGAC

CCTCATCCAACAGCCCAGAAAAGCTATTGGAGCTCATCCGGGCTGGGATGCGGGTCGTGCGTATGAACTTCTCTCACGGC
