# Supplementary material for: Metabolomic analysis of Drosophila melanogaster larvae lacking pyruvate kinase
Source: G3 (Bethesda). 2023 Oct 4;14(1):jkad228. doi: 10.1093/g3journal/jkad228 (PMC10755183; doi:10.1093/g3journal/jkad228)
Supplement: jkad228_Supplementary_Data [file jkad228_supplementary_data.zip › Supplemental_Material_Legends_G3-2023-404572.docx]

**SUPPLEMENTAL FIGURE LEGENDS**

**Figure S1. *Pyk* mRNA transcript levels are significantly reduced in *Pyk* mutant larvae.** Total RNA from stage *w^1118^; Pyk^prec^* control larvae and *w^1118^; Pyk^60/61^* mutant larvae were analyzed by northern blot hybridization to detect transcripts encoding Pyk, CG18596, and Polr3F. Hybridization to detect *rp49* mRNA is included as a loading control.

**Figure S2. A comparison of sequence similarities between Pyk homologs.**  A heatmap of pairwise sequence identities extracted from the ensemble MSA. Species abbreviations: Aa, *A*. *aegypti;*Dm, *D. melanogaster*; Hs, *H. sapiens*; Mm, *M. musculus*; Ce, *C. elegans*; Dr, *D.* *rerio, Dd, D*. *discoideum.* See Table S1 for a list of isoforms used in this analysis.

**­­**

**Figure S3. A comparison of the metabolomic data from *Pyk^23/31^* mutant and *Pyk^21/+^* control samples using Partial Least Squares Discriminant Analysis (PLS-DA).** Targeted metabolomics data from Table S2 was analyzed using PLS-DA. Analysis was conducted using Metaboanalyst 5.0.

**Figure S4.** **A comparison of the metabolomic data from *Pyk^60/61^* mutant and *Pyk^prec^* control samples using Partial Least Squares Discriminant Analysis (PLS-DA).** Targeted metabolomics data from Table S3 was analyzed using PLS-DA. Analysis was conducted using Metaboanalyst 5.0.

**Figure S5.  RNA-seq Principal Component Analysis and Correlations of Experimental Replicates.**  (A) PCA biplot of principal component 1 (PC1) versus PC2 for the top 5,000 genes by variance, with replicates labeled.  (B) Spearman correlation plot of the top 5,000 genes by variance after filtering out genes with either low/no expression.

**Figure S6. Expression of intestinal lipases and proteases are upregulated in *Pyk* mutants.** Heatmaps depicting the expression of significantly up-regulated genes in the GO categories “lipid metabolic process” and “proteolysis”. These genes are grouped by enzymatic function, with the increased fold-change in gene expression represented by a color gradient (log2-fold change in *Pyk^23/31^* mutants compared to *Pyk^23/+^* controls).

**SUPPLEMENTAL FILE TEXT**

**File S1. Sequence of the *Pyk^61^* deletion.** The sequence removed by *Pyk^61^* deletion is listed along with genomic coordinates (*Drosophila melanogaster* reference genome release 6.53).

**SUPPLEMENTAL TABLE LEGENDS**

**Table S1.** Protein isoform information for the Pyk orthologs used to conduct phylogenetic analysis (see Figure 1 and Figure S2).

**Table S2.** Metabolomic analysis of *Pyk^23/31^* mutants as compared with *Pyk^23/+^* controls. All values normalized to sample mass and a d-4-succinic acid standard. n=6 samples per genotype; 25 mid-L2 larvae per sample.

**Table S3.** Metabolomic analysis of imprecise excision *Pyk* mutants as compared with precise excision controls. All values normalized to sample mass and a d-4-succinic acid standard. n=8 samples per genotype; 25 mid-L2 larvae per sample.

**Table S4.** RNA-seq results comparing gene expression between *Pyk^23/31^* mutants and *Pyk^23/+^*.

**Table S5.** Genes encoding *Drosophila* Pyk homologs do not exhibit elevated expression in *Pyk* mutants. Data is extracted from Table S4.

**Table S6.** GO analysis of significantly mis-regulated genes from the RNAseq data represented in Table S4 using GOrilla. The enrichment value is defined as (b/n)/(B/N), where N is total number of genes, B is the total number of genes associated with a specific GO term, n is the number of genes significantly down- or upregulated in *Pyk* mutant larvae, b is the overlap between genes in the GO category and the down- or upregulated genes in *Pyk* mutant larvae.

**Table S7.** Analysis of RNA-seq data from Table S4 using the Preferred tissue (modEncode RNA_seq) tool in PANGEA.
